# CAAP48, a New Sepsis Biomarker, Induces Hepatic Dysfunction in an *in vitro* Liver-on-Chip Model

**DOI:** 10.3389/fimmu.2019.00273

**Published:** 2019-02-25

**Authors:** Nancy Blaurock-Möller, Marko Gröger, Fatina Siwczak, Julia Dinger, Diana Schmerler, Alexander S. Mosig, Michael Kiehntopf

**Affiliations:** ^1^Department of Clinical Chemistry and Laboratory Medicine, Jena University Hospital, Jena, Germany; ^2^Centre for Sepsis Control and Care (CSCC), Jena University Hospital, Jena, Germany; ^3^Institute of Forensic Medicine, Jena University Hospital, Jena, Germany

**Keywords:** alpha-1antitrypsin, serpin A1, systemic inflammation, sepsis, liver dysfunction

## Abstract

Sepsis is a leading cause of mortality in the critically ill, characterized by life-threatening organ dysfunctions due to dysregulation of the host response to infection. Using mass spectrometry, we identified a C-terminal fragment of alpha-1-antitrypsin, designated CAAP48, as a new sepsis biomarker that actively participates in the pathophysiology of sepsis. It is well-known that liver dysfunction is an early event in sepsis-associated multi-organ failure, thus we analyzed the pathophysiological function of CAAP48 in a microfluidic-supported *in vitro* liver-on-chip model. Hepatocytes were stimulated with synthetic CAAP48 and several control peptides. CAAP48-treatment resulted in an accumulation of the hepatocyte-specific intracellular enzymes aspartate- and alanine-transaminase and impaired the activity of the hepatic multidrug resistant-associated protein 2 and cytochrome P450 3A4. Moreover, CAAP48 reduced hepatic expression of the multidrug resistant-associated protein 2 and disrupted the endothelial structural integrity as demonstrated by reduced expression of VE-cadherin, F-actin and alteration of the tight junction protein zonula occludens-1, which resulted in a loss of the endothelial barrier function. Furthermore, CAAP48 induced the release of adhesion molecules and pro- and anti-inflammatory cytokines. Our results show that CAAP48 triggers inflammation-related endothelial barrier disruption as well as hepatocellular dysfunction in a liver-on-chip model emulating the pathophysiological conditions of inflammation. Besides its function as new sepsis biomarker, CAAP48 thus might play an important role in the development of liver dysfunction as a consequence of the dysregulated host immune-inflammatory response in sepsis.

## Introduction

Sepsis is the leading cause of mortality in the critically ill characterized by life-threatening organ dysfunctions due to dysregulation of the host response to infection (1). Although important advances have been made in many areas of clinical medicine, both diagnosis and treatment of sepsis continue to be one of the greatest challenges of modern medicine worldwide. Significant reduction of sepsis mortality requires timely diagnosis and identification of patients at risk as well as early and causative therapy.

In a previous study, using mass spectrometry, we identified a proteolytic fragment of alpha-1-antitrypsin (AAT), designated CAAP48 (C-terminal peptide of alpha-1-antitrypsin with a mass of 4.8 kDa), as a potential sepsis biomarker (2). The peptide was present in higher concentrations in sepsis patients compared to controls. We hypothesized that AAT is cleaved during sepsis by infection specific proteases leading to a release of CAAP48. AAT, a member of the serpin superfamily, is an acute phase protein and a circulating protease inhibitor that is known to down regulate host immune response (3). In contrast, CAAP48, as well as several variants of this C-terminal AAT-fragment [e.g., C-36 peptide, virus inhibitory peptide (VIRIP), cancer-associated, SCM-recognition, immune defense-suppressing, and serine protease-protecting (CRISPP) peptide, short piece of alpha 1-antitrypsin (SPAAT)] associated with certain pathological conditions, have pro-inflammatory immune modulating functions (4–8). Particularly, CAAP48 has been shown to activate neutrophils, induce neutrophil chemotaxis, pro-inflammatory cytokines, and the release of reactive oxygen species (ROS) (2). Besides CAAP48, a frequent SNP-variant (rs1303) occurs, designated CAAP47 (C-terminal peptide of alpha-1-antitrypsin with a mass of 4.7 kDa due to an E>D substitution) that shows less inflammatory activity compared to CAAP48 (2).

The liver is among the first organs affected during sepsis and liver failure is associated with a poor sepsis outcome (9, 10), presumably due to its central metabolic role and involvement in the regulation of the host-response to infection. During sepsis the hepatocellular inflammatory response leads to an increase in the synthesis of acute-phase proteins, such as AAT (11), as well as to profound changes in the coagulation system, glucose metabolism (11) and the biotransformation capacity, reflected by e.g., a reduced activity of the cytochrome P450 (CYP) system. Therefore, the elimination of xenobiotics and endobiotics is conspicuously impaired (11, 12).

To investigate whether CAAP48 might play a role in sepsis related organ dysfunction of the liver we first considered to study the pathophysiological function of CAAP48 and the SNP variant CAAP47 in a sepsis mouse model. However, the murine AAT sequences differs compared to human AAT, particularly at the cleavage site, thus a sepsis mouse model might be not appropriate for investigating CAAP47/48 role in liver dysfunction. Moreover, overexpression of human AAT in a mouse model results in reduced lethality (13), while administration of a human AAT variant in a primate sepsis model leads to exacerbation and septic shock, probably due to the high concentrations of cleaved AAT, which has been shown to induce a strong immune response (14). Thus, we decided to study the pathophysiological effects of CAAP47/48 on liver function in an well-established *in vitro* liver-on-chip model, which emulates the human liver microphysiology by integration of the four major human liver cell types in microfluidically perfused biochips (15–17), in order to establish a link between CAAP48 and sepsis-associated liver dysfunction.

## Materials and Methods

### Peptides

The peptides were purchased from ProteoGenix SAS (Schiltigheim, France). The lyophilized peptides were dissolved in sterile PBS w/o (without Ca and Mg) at a concentration of 400 μM and stored at −20°C. Peptide stock solutions were tested negative for endotoxin contamination using the Limulus Color KY test from FUJIFILM Wako Pure Chemical Corporation according to the manufacturer's protocol (<0.0002 EU/ml; detection limit of the test kit). For each experiment, fresh aliquots were thawed. The synthetic peptides were used at an assay concentration of 40 μM, based on results from a previous study, in which optimal effects of the peptides on immune cells with minimal influence on cell viability could be observed at 40 μM (2). Table 1 gives an overview about the different peptides used.

**Table 1:** AAT peptides.

**Peptide**	**Sequence**
CAAP48	H-LEAIPMSIPPEVKFNKPFVFLMIEQNTKSPLFMGKVVNPTQK-OH
CAAP47	H-LEAIPMSIPPEVKFNKPFVFLMIDQNTKSPLFMGKVVNPTQK-OH
scrambled peptide	H-KKFISPFVKMPNFVTSGPVIPQNEKFLMEQPKLMVPITLENA-OH

*The lyophilized peptides were used at an assay concentration of 40 μM*.

### Cell Culture

HepaRG hepatocytes were obtained from Biopredic International (Rennes, France) and cultured in William’s Medium E (Biochrom, Berlin, Germany) supplemented with 10% (v/v) FCS (Life Technologies, Darmstadt, Germany), 2 mM glutamine (GIBCO, Darmstadt, Germany), 50 mM hydrocortisone-hemisuccinate (Sigma-Aldrich, Steinheim, Germany), 5 μg/ml insulin (Sigma-Aldrich, Steinheim, Germany) and a mixture of 100 U/ml Penicillin/100 μg/ml Streptomycin (GIBCO, Darmstadt, Germany). Cells were seeded at 2.7 × 10^4^ cells/cm^2^ and cultured at 5% CO_2_ and 37°C for 14 days before differentiation and medium changed every 3–4 days. Cell differentiation was induced as described by Gripon et al. (18) and used up to 4 weeks. Human umbilical cord vein endothelial cells (HUVECs) were isolated from human umbilical cord veins as described by Jaffe et al. (19). HUVECs were cultured in Endothelial Cell Medium (Promocell, Heidelberg, Germany) up to passage four. LX-2 stellate cells were kindly allocated by Scott L. Friedman (Division of Liver Disease, Mount Sinai School of Mediciner, New York City, NY, USA). LX-2 cells were cultured in Dulbecco's Minimum Essential Medium (Biochrom, Berlin, Germany) containing 10% (v/v) FCS (Life Technologies, Darmstadt, Germany), 1 mM sodium pyruvate (GIBCO, Darmstadt, Germany) and 100 U/ml Penicillin/100 μg/ml Streptomycin mixture (GIBCO, Darmstadt, Germany). HUVECs were seeded at a density of 2.0 × 10^6^ cells/cm^2^. Peripheral Blood Mononuclear Cells (PBMCs) were isolated from the peripheral blood using Ficoll density gradient centrifugation as described by Mosig et al. (20). For monocyte enrichment PBMCs were seeded at 1.0 × 10^6^ cells/cm^2^ and cultured at 5% CO_2_ and 37°C and cultured in X-VIVO 15 medium (Lonza, Cologne, Germany) containing 10% (v/v) autologous human serum, 10 ng/ml human granulocyte macrophage colony-stimulating factor (ProTech, Hamburg, Germany) and a mixture of 100 U/ml Penicillin and 100 μg/ml Streptomycin (GIBCO, Darmstadt, Germany). After 3 h the cells were washed with X-VIVO 15 medium and adherent monocytes were cultivated for 24 h in X-VIVO 15 medium. HUVECs and PBMCs were isolated from blood of healthy volunteers in accordance with Helsinki Declaration. Blood donors were informed about the aim of the study and written informed consent was obtained. The study and all protocols were approved by the local ethics committee (approval number 3939-12/13).

### Biochips, Cell Culture in the Biochips, and Stimulation With AAT-peptides

MOTiF biochips were manufactured from microfluidic ChipShop GmbH (Jena, Germany) as described by Raasch et al. (15). The design of the MOTiF biochips as well as the cell culture in the biochips is specified by Gröger et al. (17). HepaRG cell layer was incubated with 40 μM synthetic peptide (CAAP47/48 and scrambled peptide) and without peptide (negative control) for 72 h at 5% CO_2_ and 37°C. Medium was exchanged every 24 h. All experiments shown were performed in the liver-on-chip model.

### AlamarBlue Assay

AlarmaBlue (Resazurin Sodium Salt, Sigma-Aldrich Chemie GmbH, Steinheim) contains the cell permeable, non-toxic, and weakly fluorescent indicator dye resazurin. It is an indicator for metabolic cell function because it undergoes colorimetric change in response to cellular metabolic reduction. HepaRG cell layer was incubated with serum free Williams E medium containing 10% (of the sample volume) AlamarBlue reagent for 2 h at 5% CO_2_ and 37°C. The resulting fluorescence was read out on a Tecan Safire2TM plate reader (Tecan Group Ltd., Männedorf, Switzerland) at 570 and 690 nm (reference) emission wavelength. All experiments were performed in duplicates.

### Immunofluorescence Staining

Cells were fixed with 4% paraformaldehyde (PFA) for 10 min at room temperature. Staining was performed using antibodies against: ApoB (Santa Cruz, Heidelberg, Germany), VE-Cadherin (BD Biosciences, Heidelberg, Germany), MRP2 (Cell Signaling, Leiden, Netherlands), and ZO-1 (Life Technologies, Darmstadt, Germany) and secondary antibodies goat-anti-mouse-Cy3 (Santa Cruz, Heidelberg, Germany), goat-anti-rabbit-AlexaFluor488 (Life Technologies, Darmstadt, Germany), AlexaFluor633 phalloidin (Life Technologies, Darmstadt, Germany), and DAPI (Life Technologies, Darmstadt, Germany). Samples were enclosed into fluorescent mounting medium (Dako, Hamburg, Germany). Cells were analyzed using an AXIO Observer Z1 fluorescence microscope with Apotome 2 extension (Carl Zeiss AG, Jena, Germany). The software FiJi (GNU General Public License) was used for image analysis.

### DY-635 Staining and CDF-DA Assay

The MRP2 activity was analyzed by incubation of the HepaRG cell layer in serum free Williams E medium without phenolred (GIBCO, Darmstadt, Germany) containing 5 μM 5(6)-carboxy-2′,7′-dichlorofluorescein diacetate (CDF-DA) (Sigma-Aldrich, Steinheim, Germany) or 10 μM 2-tert-Butyl-4-{(E)-3-[1-(5-carboxy-pentyl)-3,3-dimethyl-5-sulfo-1,3-dihydro-indol-(2Z)-ylidene]-propenyl}c-7-diethylamino-1-benzopyranylium (DY-635) (Dyomics, Jena, Germany) at 37°C. After 15 min the cell layer was incubated with William’s medium E without phenolred for 30 min. Subsequently, cells were analyzed using an AXIO Observer Z1 fluorescence microscope with Apotome 2 extension (Carl Zeiss AG; Jena, Germany). The software FiJi (GNU General Public License) was used for image analysis.

### Analysis of CYP3A4 Activity

After stimulation with AAT-peptides, liver-on-chip were incubated with serum-free William's medium E containing 3 μM Midazolam (Rotexmedica, Trittau, Germany). The samples were precipitate and concentrated and afterwards analyzed on a LC-20 liquid chromatography system (Shimadzu, Japan) connected to an ABSciex QTrap4000 tandem mass spectrometer (ABSciex, USA) as previously described (16, 17).

### Quantification of Secreted Proteins

Supernatants of the upper chamber were collected and stored at −80°C until analysis. Concentrations of the cytokines VCAM-1 and ICAM-1 were determined by Bio-Plex assay (Pro Human Cytokine 2-plex Assay (VCAM, ICAM), BioRad Laboratories, Munich, Germany), according to the manufacturer's protocol. Cytokines IL-1β, TNFα, IL-6, and IL-10 were identified using CBA assay (BD Biosciences, Heidelberg, Germany) according to the manufacturer's instructions (enhanced sensitivity flex set for IL-1β and TNFα; standard CBA flex sets for IL-6, IL-10, and MCP-1). Samples were analyzed on a FACS Canto II flow cytometer (BD Biosciences, Heidelberg, Germany). The software FlowJo 7.6.4 (BD Biosciences, Heidelberg, Germany) was used for analysis.

### Measurements of AST and ALT

The concentrations of the laboratory parameters were measured within the cell culture supernatants of the lower chamber according to the manufacturer's protocol using the Abbott Architect ci8200 Integrated System (Abbott Laboratories, Abbott Park, IL, USA).

### Statistics and Data Analysis

**Figure 1:**
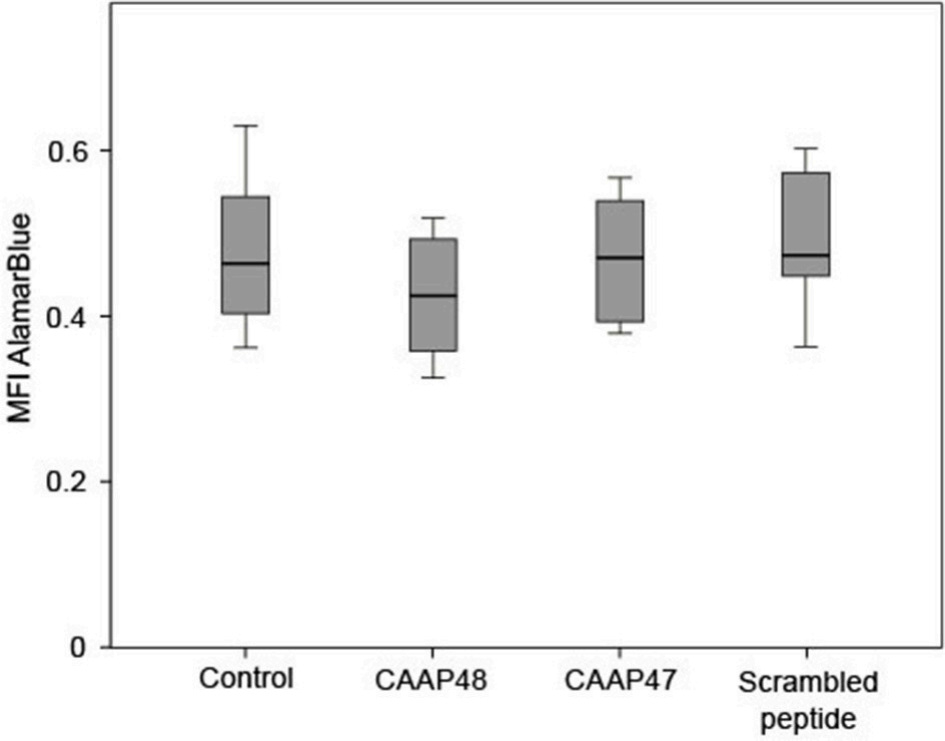
Viability of HepaRG cells after stimulation with the AAT peptides. The AlamarBlue® dye is a redox indicator and the assay is based on the ability of metabolically active cells to convert the reagent into a fluorescent and colorimetric indicator. Damaged and non-viable cells have lower innate metabolic activity and generate a proportionally lower signal. The HepaRG cells embedded within the liver-on-chip model were incubated with 40 μM of the AAT peptides for 72 h and the color change of the redox indicator resazurin was determined on the Tecan Safire2™ at 570 and 600 nm (reference). Results of six independent experiments are shown. Control (0.463, [0.403–0.57]), CAAP48 (0.424, [0.342–0.493]), CAAP47 (0.47, [0.388–0.542]), Scrambled peptide (0.471, [0.446–0.581]).

**Figure 2:**
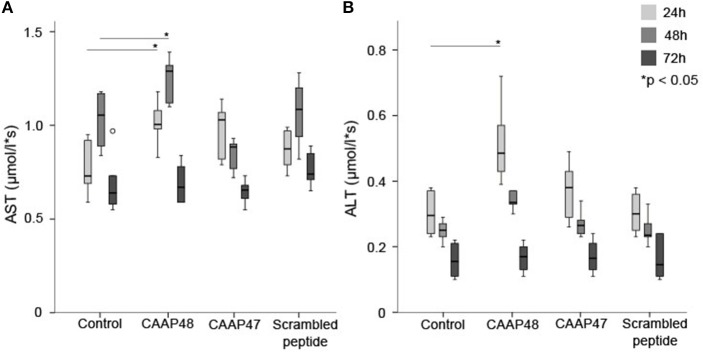
Release of the intracellular enzymes AST and ALT. The laboratory parameters aspartate-transaminase (AST, **A**) and alanine-transaminase (ALT, **B**) were detected in the cell culture supernatants of the hepatic layer of the liver-on-chip model. Results of six independent experiments are shown. **(A)**: Control 24/48/72h (0.73, [0.59–0.93]/1.06, [0.84–1.17]/0.64, [0.57–0.97]), CAAP48 24/48/72h (1.01, [0.89–1.18]/1.29, [1.12–1.36]/0.67, [0.59–0.84]), CAAP47 24/48/72h (1.03, [0.79–1.09]/0.89, [0.77–0.92]/0.66, [0.55–0.73]), Scrambled peptide 24/48/72h (0.88, [0.76–0.98]/1.09, [0.82–1.2]/0.74, [0.65–0.89]). **(B)** Control 24/48/72h (0.3, [0.23–0.38]/0.25, [0.2–0.27]/0.16, [0.11–0.22]), CAAP48 24/48/72h (0.49, [0.39–0.72]/0.34, [0.31–0.37]/0.17, [0.11–0.2]), CAAP47 24/48/72h (0.38, [0.26–0.49]/0.27, [0.24–0.31]/0.17, [0.13–0.23]), Scrambled peptide 24/48/72h (0.3, [0.23–0.37]/0.24, [0.2–0.33]/0.15, [0.1–0.24]).

**Figure 3:**
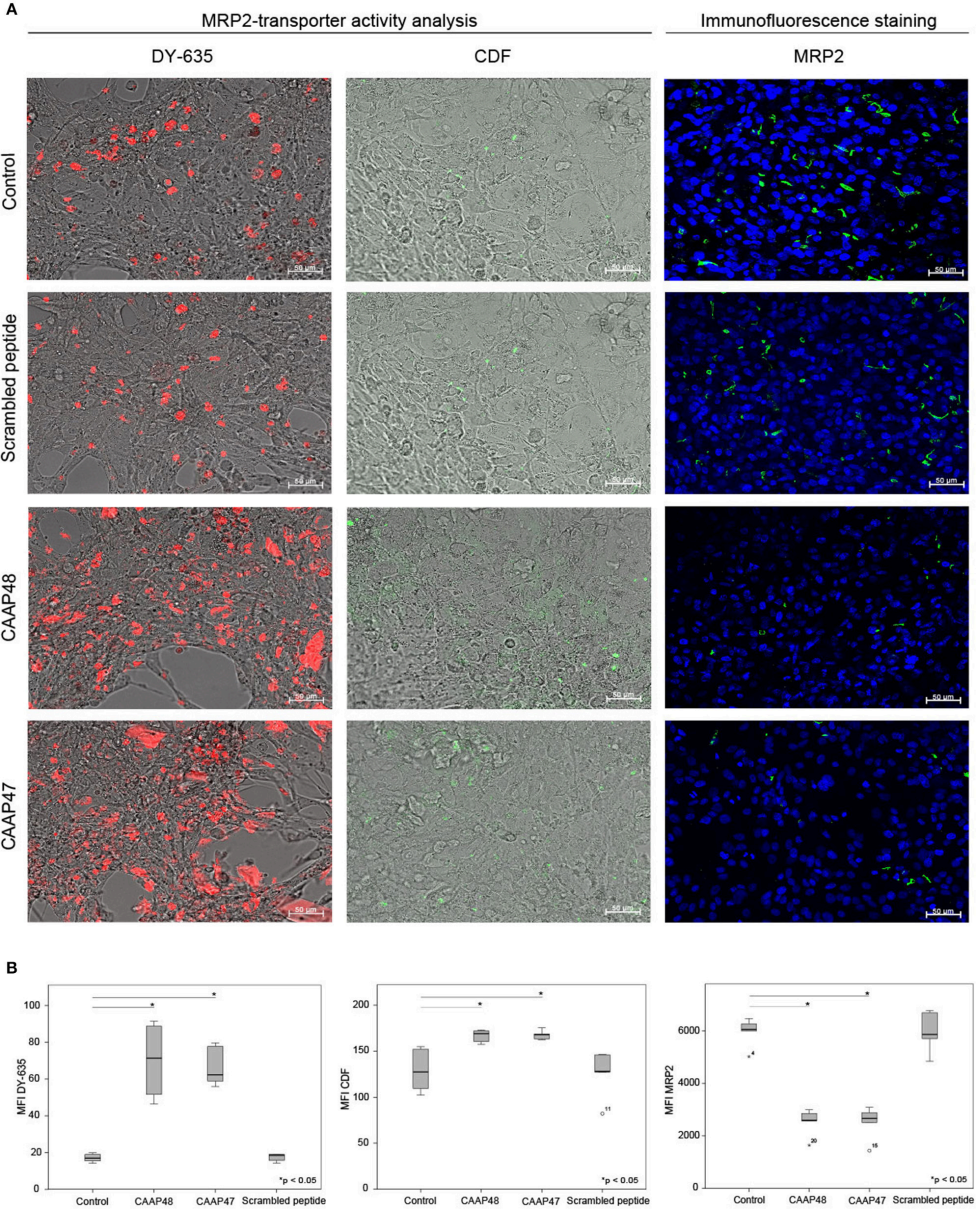
MRP2-transporter activity analysis in the hepatic layer of the liver-on-chip model. **(A)** Immunostaining for multidrug resistant-associated protein 2 (MRP2, green) and detection of DY-635 (red) and CDF secretion (green) in the hepatic layer. Nuclei are stained with DAPI (blue). Data of one exemplary experiment out of six independent experiments is shown. Scale bar: 50 μm. **(B)** Computational analyses of mean fluorescence intensities (MFI) of at least 20 regions of interest (ROI) per tested condition of the respective protein using random field analysis. Results of six independent experiments are shown. DY635/CDF/MRP2: Control (16.9, [15.6–19.2]/127.6, [109.8–152.3]/6,150, [6,000–6,450]), CAAP48 (71.3, [54.8–87.9]/ 169.1, [160.7–172.3]/ 2,650, [2,600–3,000]), CAAP47 (62.2, [60.5–78.3]/168.1, [163.6–168.7]/2,750, [2,550–3,200]), Scrambled peptide (18.5, [14.5–19.0]/ 128.3, [127.5–146.1]/6,000, [4,900–6,800]).

**Figure 4:**
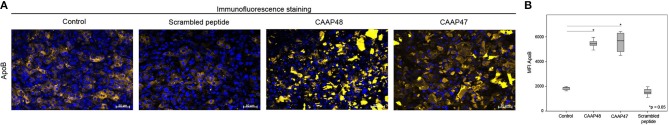
Immunofluorescence staining of the lipoprotein ApoB in the hepatic layer of the liver-on-chip model. **(A)** Immunofluorescence staining of apolipoprotein B (ApoB, yellow) in the hepatic layer. Nuclei are stained with DAPI (blue). Data of one exemplary experiment out of six independent experiments is shown. Scale bar: 50 μm. **(B)** Computational analyses of mean fluorescence intensities (MFI) of at least 20 regions of interest (ROI) per tested condition of the respective protein using random field analysis. Results of six independent experiments are shown. Control (18846, [16685–19585]), CAAP48 (54411, [51883–57389]), CAAP47 (52463, [44992–59897]), Scrambled peptide (14787, [13910–17440]).

**Figure 5:**
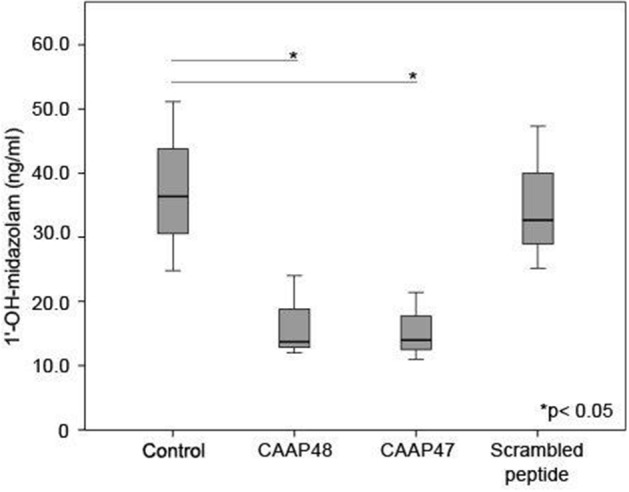
CYP3A4 activity without stimulation and in presence of the AAT peptides. To determine the CYP3A4 activity, the conversion of midazolam to 1′-hydroxy-midazolam (1′-OH-midazolam) was measured by LC-MS/MS. Results of three independent experiments are shown. Control (36.4, [26.4–51.1]), CAAP48 (13.7, [12–24]), CAAP47 (14, [11–21.4]), Scrambled peptide (32.7, [25.2–47.3]).

**Figure 6:**
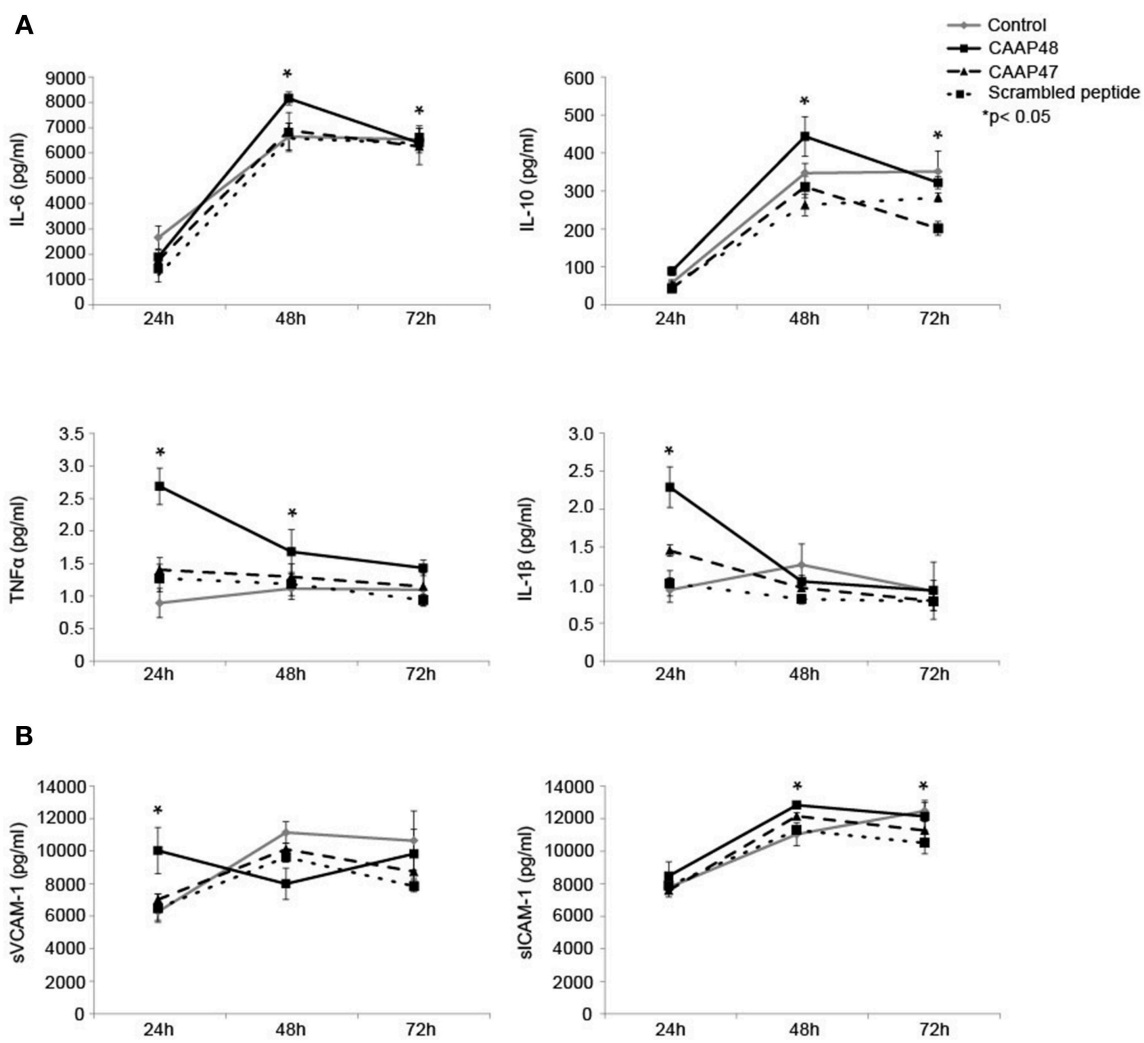
Release of cytokines and adhesion molecules in the liver-on-chip model stimulated with CAAP48. **(A)** Cytokine concentrations of IL-1β, IL-6, TNFα, and IL-10 within the supernatants of the vascular layer were determined by flow cytometry using a bead-based multiplex immunoassay. **(B)** The release of sVCAM-1 and sICAM-1 within the supernatants of the vascular layer were examined by Bio-Plex assay. Results of six independent experiments are shown.

For each experiment at least three independent replicates were analyzed. Statistical analysis was performed with SPSS 21 (IBM, USA) or GraphPad Prism 6.05 (GraphPad Software, La Jolla, CA, USA). For analysis of statistical significance one-way ANOVA with Bonferroni *post-hoc* test (homogeneity of variances) or Games-Howell *post-hoc* test (no variance homogeneity) (Figures 1, **3–5**) or two-way ANOVA with Tukey's multiple comparisons test (**Figures 2, 6**) have been used. The level of significance (*p*-value) was 0.05. Box plots were generated according to standard definitions: bold lines depict median values, boxes interquartile ranges (IQR) between upper (75th) and lower (25th) quartile and whiskers extreme values within the 2.5-fold IQR around the median; values outside this range are marked as outliers (21). Dots present mild outliers and asterisks extreme outliers. The boxes and whiskers were described as (median, [CI95%]) in each figure legend.

## Results

### The AAT-peptides Do Not Have Cytotoxic Effects on HepaRG

Initially potential cytotoxic effects of the AAT peptides on isolated HepaRG cells were excluded by measurement of mitochondrial metabolic activity in the liver-on-chip model. HepaRG treated with the peptides for up to 72 h showed no significant differences compared to the unstimulated control (Figure 1).

### Hepatic Enzyme Expression Is Modulated by CAAP48

The hepatocellular tissue injury was assessed by measurement of the release of hepatocyte-specific intracellular enzymes aspartate-transaminase (AST) and alanine-transaminase (ALT) in the liver-on-chip. Stimulation with CAAP48 resulted in accumulation of AST (Figure 2A) and ALT (Figure 2B) in the cell culture supernatants of the hepatic layer. In contrast, incubation of the hepatocytes with CAAP47 or scrambled peptide did not result in an increased release of AST/ALT (Figures 2A,B).

### CAAP48 Regulate Hepatic Protein Expression

*In vivo*, liver dysfunction is associated with a reduced expression of the hepatobiliary transporter multidrug resistant-associated protein 2 (MRP2) resulting in cholestasis (22). After 72 h of CAAP47/48 treatment, MRP2-expression was reduced by about 50% in comparison with the untreated control and incubation with scrambled peptide. To assess MRP2 transporter activity we used the dyes DY-635 and 5(6)-carboxy-2′, 7′-dichlorofluorescein (CDF), which are specifically released into bile canaliculi via MRP2 (17, 23, 24). We measured the dye secretion efficiency of both dyes to examine the effect of CAAP47/48 stimulation on MRP2-activity. In response to CAAP47/48 stimulation, a reduction of secretion efficiency and accumulation of DY-635 and CDF within the cytoplasm was observed. In contrast, incubation with the scrambled peptide had no effect on MRP2 transporter activity (Figures 3A,B). Additionally, we observed an altered expression and localization of apolipoprotein B (ApoB) (Figures 4A,B), a transporter protein mainly involved in lipoprotein metabolism (25). As shown in Figure 4A, the ApoB signal seems to spread across cell borders, leading to the assumption of hepatocyte fusion in response to CAAP47/48 treatment that was not observed in response to stimulation with the scrambled peptide.

### CAAP47/48 Affects the Drug-Metabolizing Capacity of Hepatocytes

Cytochrome P450 enzymes (CYPs) are the most important enzymes involved in drug metabolism of the liver. In this context, CYP3A4 is involved in the metabolism of more than 50% of prescribed drugs (26). To investigate whether CAAP47 or CAAP48 effect drug-metabolizing capacity of hepatocytes we measured the CYP3A4-depended metabolization of midazolam based on the formation of 1-hydroxy-midazolam (1-OH-midazolam) (27) and found a 50% decreased level of 1-OH-midazolam in response to CAAP47 and CAAP48 compared to the scrambled peptide (Figure 5).

### CAAP48 Specifically Induces the Secretion of Cytokines

Pro-inflammatory cytokines, such as interleukin (IL)-6, IL-1β, and tumor necrosis factor (TNF) α have been shown to contribute to sepsis-associated hepatocellular dysfunction (28). We thus analyzed the secretion of these cytokines in response to the cleaved AAT peptides. Stimulation with CAAP48 for 24 h increased the levels of IL-1β and TNFα whereas secretion of IL-6 and IL-10 was found increased after 48 h of CAAP48 treatment (Figure 6A). In contrast, stimulation with CAAP47 did not induce an increased release of cytokines (Figure 6A).

### Release of Adhesion Molecules in Response to CAAP48

Shedding of the endothelial adhesion molecules vascular adhesion molecule 1 (VCAM-1) and intercellular adhesion molecule 1 (ICAM-1) has been shown to be an important process in controlling leucocyte migration and to enhance cytokine release during sepsis in the liver (29). Furthermore, soluble fragments of VCAM-1 and ICAM-1 are correlated with the severity of sepsis (30). We thus measured the release of soluble VCAM-1 (sVCAM-1) and soluble ICAM-1 (sICAM-1) peptides from the cell surface in response to CAAP48-treatment. sVCAM-1 levels increased after 24 h and sICAM-1 levels after 48 and 72 h of stimulation (Figure 6B). Interestingly, in contrast to CAAP48, CAAP47 stimulation did not result in an increased release of these adhesion molecules (Figure 6B).

### CAAP48 Modulates Endothelial Protein Expression

**Figure 7:**
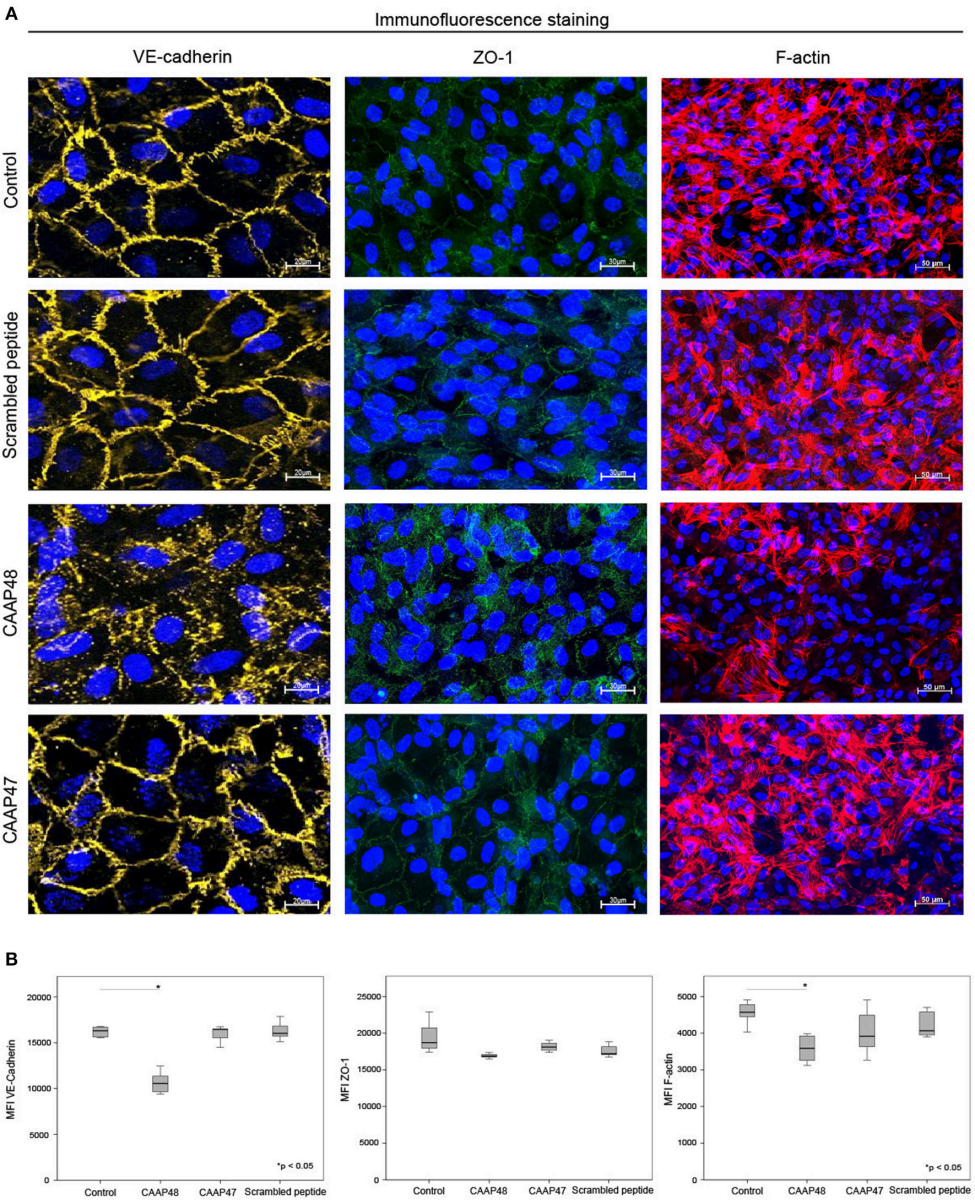
Immunofluorescence staining of endothelial proteins. **(A)** Expression of VE-cadherin (yellow), ZO-1 (green) and F-actin (red) at the vascular layer of the liver-on-chip model. Representative results of six independent experiments are shown. Scale bars 20 μm (VE-cadherin), 30 μm (ZO-1), 50 μm (F-actin). **(B)** Computational analyses of MFI of at least 20 ROI per tested condition of the respective protein using random field analysis. Results of six independent experiments are shown. VE-cadherin/ZO-1/F-Actin: Control (16302, [15550–16643]/18705, [17519–21225]/45716, [44318–48369]), CAAP48 (10562, [9561–11287]/16884, [16771–17111]/35908, [32545–39327]), CAAP47 (16458, [15837–16471]/18108, [17377–19042]/39179, [35624–47113]), Scrambled peptide (16017, [15789–16706]/17194, [17085–18375]/40668, [39056–46013]).

Loss of the endothelial barrier function is an important aspect in the pathogenesis of organ dysfunction during sepsis (31). We therefore analyzed proteins important for maintenance of endothelial barrier function. The F-actin filament system and junctional proteins, like VE-cadherin or the zonula occludens-1 (ZO-1) protein, are known to play a crucial role in maintaining endothelial barrier function (32–36). We thus analyzed the expression levels of these proteins in the vascular layer of the liver-on-chip. Within 3 days of CAAP48-stimulation the expression levels of VE-cadherin and F-actin were markedly reduced by ~35 and 25%, respectively. Furthermore, ZO-1 was disorganized in endothelial cells. Treatment with CAAP47 or the scrambled peptide however did not affect expression of the junctional proteins or assembly of actin filaments (Figures 7A,B).

## Discussion

The purpose of the present study was to investigate the pathophysiological function of CAAP48 in an *in vitro* liver-on-chip model of the human sinusoid. The model was recently demonstrated to be an appropriate tool to investigate inflammation-related liver dysfunction with changes at the molecular and cellular level that closely resemble pathophysiological alterations of liver function observed in the murine sepsis model of peritoneal contamination and infection (PCI) as well as clinical observations in human suffering from sepsis (17).

Our data show that CAAP48 is a strong pro-inflammatory peptide that triggers hepatocellular dysfunction by down regulation of hepatic transport proteins, disruption of the structural and functional integrity of tight junction proteins as well as changes of the cytoskeleton.

Liver dysfunction is an early event in sepsis (37) and associated with the release of AST and ALT in plasma, with a maximum 24 h after the onset of septic shock (11). After 2–3 days transaminase levels typically decline and reach normal values within 15 days (11). We observed a similar time-course in response to CAAP47/48 in the liver-on-chip model, confirming a direct and detrimental impact of these peptides on liver cells equivalent to liver damage in sepsis.

CAAP48 stimulation further caused the release of pro-inflammatory cytokines such as IL-1β, IL-6, and TNFα. *In vivo*, these cytokines could contribute to injury of endothelial cells and hepatocytes during sepsis (37). TNFα directly stimulates hepatocytes to produce IL-6, the major mediator of acute phase response, which is known to be increased in sepsis patients and associated with a higher risk of death (38, 39). TNFα and IL-1β activate endothelial cells and induce an increased expression of the adhesion molecules ICAM-1 and VCAM-1 (40, 41). These molecules interact with circulating leukocytes and control adhesion and invasion of immune cells into inflamed liver tissue, thus contributing to neutrophil-mediated liver injury (37). In addition to the transmembrane form of these endothelial cell adhesion molecules (CAMs), soluble forms of CAMs (sCAMs) were described (29). The release of these sCAMs by shedding of ICAM-1 and VCAM-1 were demonstrated to be induced by CAAP48. Up-regulation and shedding of these CAMs is in line with observations that enrichment of sCAM fragments positively correlated with the severity and outcome of sepsis as well as other inflammatory diseases (30). Our results thus suggest that CAAP48 could actively participate in the progression of hepatocellular dysfunction by triggering the shedding of VCAM-1 and ICAM-1.

In addition to the production of pro-inflammatory cytokines, the response of the liver to sepsis involves the induction of anti-inflammatory mediators (28). Clinical studies in adults have reported increased IL-10 plasma levels in patients suffering from sepsis that are correlated with an increase in plasma levels of pro-inflammatory cytokines (42). In agreement with these findings, in the liver-on-chip model IL-6 and IL-10 concentrations were found to be increased after CAAP48 treatment, indicating that CAAP48 contributes to the regulation of cytokine release during sepsis.

Pro-inflammatory cytokines (e.g., TNFα and IL-1β) were also shown to modulate the expression level of transporter proteins within the liver (43). In particular, the excretory function of the liver is often impaired in patients suffering from sepsis due to a disturbed expression and localization of the membrane transporter protein MRP2 at the bile canaliculi. CAAP47/48 stimulation significantly reduced MRP2-expression and impaired MRP2 transporter activity. This is in line with observations in a septic rat model and *in vivo* data, were reduced MRP2 activity leads to impaired bile acid transport and secretion (44, 45), as well as disturbed bile acid conjugation and cholestasis, respectively, a clinical symptom frequently observed in the course of sepsis (24, 44). However, MRP2 is not only responsible for excretion of endogenous substrates, but also for metabolization of xenobiotics including antibiotics (e.g., ceftriaxone, ampicillin) that are typically used in sepsis therapy (46). In septic patients with liver failure, accumulation of toxic substances due to restricted metabolic capacity of the liver often leads to subsequent organ failure (47). Consequently, regular drug uptake into hepatocytes, intracellular drug metabolism, and subsequent excretion of drug metabolites into bile is an important determinant for the pharmacokinetics and pharmacodynamics of orally administered drugs (48).

Apart from impaired hepatobiliary transport, restricted activity in phase I detoxification machinery, including cytochrome P450 enzymes, during sepsis contributes to further accumulation of potentially toxic substances in hepatocytes that have to be excreted via bile secretion (37, 45, 49–51). In the liver-on-chip model we observed a reduced formation of 1-OH-midazolam upon CAAP47 and CAAP48 stimulation, which demonstrates a reduced CYP3A4 activity. In the PCI mouse model of sepsis the expression and activity of the murine homolog for CYP3A4 was found primarily diminished in acute phase response to sepsis, but increased during recovery from sepsis in surviving animals (17). In human, a reduced P450 CYP activity is associated with a poor outcome in sepsis and the development of multiple organ failure (MOF) (12, 49, 52).

During sepsis, inflammatory mediators lead to dysregulation of the apical junctional complex formation, which results in loss of the endothelial barrier function, a major pathogenic mechanism supporting MOF (53). The results presented, demonstrate that CAAP48 evolves its pathophysiological function also by disturbance of the endothelial barrier maintenance. CAAP48 effects VE-cadherin as well as ZO-1 expression and its cellular localization. In addition, actin cytoskeleton fibers were found to be disassembled in response to CAAP48. VE-cadherin in endothelial cells improves tightness of the endothelial barrier and promotes endothelial growth, motility, and aggregation (32, 36). Accordingly, inhibition of VE-cadherin results in decreased cell-cell adhesion with increased permeability of the endothelial layer. ZO-1 connects tight junction proteins to the actin cytoskeleton and is centrally involved in the organization and maintenance of endothelial integrity. Desai et al. reported that ZO-1 has been involved in a structural disarrangement of the actin cytoskeleton in response to IL-6, thus contributing to increased endothelial permeability (35).

Zhang et al. reported that inflammation induced by LPS causes cytoskeleton disruption and restricts microvascular endothelial barrier integrity through activation of NOS, RhoA, and NF-κB (54). Although speculative, it might be possible that CAAP48, similar to LPS, mediates the observed effects via TLR4. Preliminary results suggest that inhibition of TLR4 on monocytes by TAK-242, a small-molecule-specific inhibitor of TLR4 signaling, inhibits the CAAP48-induced IL-6 production (data not shown). However, whether these results could be transferred to hepatocytes remains to be elucidated and needs to be systematically addressed in follow-up studies.

A further limitation of our study is that the liver-on-chip model could not fully reflect all aspects of human physiology triggered by CAAP48 since it represents a simplified model that only allows observation of the principle modes of action of the peptides investigated. Thus, further studies are needed to confirm the role of CAAP48 in sepsis associated hepatic injury/dysfunction in the clinical setting.

In conclusion, our findings demonstrate that CAAP48 is a strong pro-inflammatory peptide that triggers pathophysiological alterations including loss of hepatic transport activity and impairment of endothelial integrity associated with cytoskeletal rearrangements. This suggests that CAAP48 not only contributes to an increased inflammatory response but also might contribute to progression of liver dysfunction. Follow-up studies are required to elucidate the detailed molecular and cellular mechanisms triggered by CAAP48 in sepsis-related liver dysfunction.

## Data Availability

All data generated during this study are available from NB-M (nancy.blaurock@med.uni-jena.de).

## Ethics Statement

This study was carried out in accordance with the recommendations of the local ethics committee of Friedrich-Schiller University Jena. All subjects gave written informed consent in accordance with the Declaration of Helsinki. All protocols were approved by the local ethics committee of Friedrich-Schiller University Jena (approval number 3939-12/13).

## Author Contributions

MK, AM, MG, DS, and NB-M: study conception and design. AM, NB-M, MG, DS, and MK: data interpretation. NB-M, MG, and FS: cell culture assays. NB-M: drafting of the manuscript. JD: mass spectrometric measurement. AM, MK, and DS: contribution of reagents, materials, and analysis tools. AM, MK, and DS: supervision of experiments. NB-M, DS, MK, AM, MG, FS, and JD: critical revision and final approval of the manuscript.

### Conflict of Interest Statement

The institution of NB-M, MG, FS, JD, DS, AM, and MK has granted patents (EP2592421, CN104204808A, EP2780719) and pending patent applications (US20140248631, JP2014533368, EP3239712, WO2017186842, EP3140390, DE102014106423, US20170226457, JP2017514524, WO2015169287). The authors declare that the research was conducted in the absence of any commercial or financial relationships that could be construed as a potential conflict of interest.

## Abbreviations

AAT: alpha-1-antitrypsin
ALT: alanine-transaminase
ApoB: apolipoprotein B
AST: aspartate-transaminase
ATP: adenosine triphosphate
CAAP: C-terminal peptide of alpha-1-antitrypsin
CAAP47/48: CAAP47 and CAAP48
CDF: 5(6)-carboxy-2′, 7′-dichlorofluorescein
CDF-DA: 5(6)-carboxy-2′, 7′-dichlorofluorescein diacetate
CRISPP: cancer-associated, SCM-recognition, immune defense-suppressing and serine protease-protecting
CYP: cytochrome P450
DY-635: 2-tert-Butyl-4-{(E)-3-[1-(5-carboxy-pentyl)-3, 3-dimethyl-5-sulfo-1,3-dihydro-indol-(2Z)-ylidene]-propenyl}c-7-diethylamino-1-benzopyranylium
FACS: fluorescence-activated cell sorting
HUVEC: human umbilical vein endothelial cells
ICAM: intercellular adhesion molecule
IL: interleukin
LPS: lipopolysaccharides
LX-2: stellate cells
MOF: multiple organ failure
MRP2: multidrug resistance-associated protein 2
NF-κB: nuclear factor ‘kappa-light-chain-enhancer’ of activated B-cells
NOS: reactive nitrogen species
PBS w/o: phosphate-buffered saline without Calcium and Magnesium
PCI: peritoneal contamination and infection
PFA: paraformaldehyde
RhoA: Ras homolog gene family member A
ROS: reactive oxygen species
SPAAT: short piece of alpha 1-antitrypsin
TLR-4: Toll-like receptor 4
TNFα: tumor necrosis factor alpha
VCAM: vascular adhesion molecule
VE-cadherin: vascular endothelial cadherin
VIRIP: virus inhibitory peptide
ZO-1: zonula occludens 1.

## References

[1] Singer M Deutschman CS Seymour CW Shankar-Hari M Annane D Bauer M The third international consensus definitions for sepsis and septic shock (Sepsis-3). J Am Med Assoc. (2016) 315:801–10. 10.1001/jama.2016.0287 PMC4968574 26903338

[2] Blaurock N Schmerler D Hunniger K Kurzai O Ludewig K Baier M. C-terminal alpha-1 antitrypsin peptide: a new sepsis biomarker with immunomodulatory function. Med Inflam. (2016) 2016:6129437. 10.1155/2016/6129437 27382189 PMC4921625

[3] Janciauskiene S Larsson S Larsson P Virtala R Jansson L Stevens T. Inhibition of lipopolysaccharide-mediated human monocyte activation, in vitro, by alpha1-antitrypsin. Biochem Biophys Res Commun. (2004) 321:592–600. 10.1016/j.bbrc.2004.06.123 15358147

[4] Cercek L Cercek B. Cancer-associated SCM-recognition, immunedefense suppression, and serine protease protection peptide. Part Isolation, I, amino acid sequence, homology, and origin. Cancer Detect Prev. (1992) 16:305–19. 1473120

[5] Johansson J Grondal S Sjovall J Jornvall H Curstedt T. Identification of hydrophobic fragments of alpha 1-antitrypsin and C1 protease inhibitor in human bile, plasma and spleen. FEBS Lett. (1992) 299:146–8. 10.1016/0014-5793(92)80234-8 1544487

[6] Niemann MA Narkates AJ Miller EJ. Isolation and serine protease inhibitory activity of the 44-residue, C-terminal fragment of alpha 1-antitrypsin from human placenta. Matrix (1992) 12:233–41. 10.1016/S0934-8832(11)80066-1 1406456

[7] Janciauskiene S Zelvyte I Jansson L Stevens T. Divergent effects of alpha1-antitrypsin on neutrophil activation, *in vitro*. Biochem Biophys Res Commun. (2004) 315:288–96. 10.1016/j.bbrc.2004.01.055 14766206

[8] Munch J Standker L Adermann K Schulz A Schindler M Chinnadurai R. Discovery and optimization of a natural HIV-1 entry inhibitor targeting the gp41 fusion peptide. Cell (2007) 129:263–75. 10.1016/j.cell.2007.02.042 17448989

[9] Wang P Ba ZF Chaudry IH. Hepatic extraction of indocyanine green is depressed early in sepsis despite increased hepatic blood flow and cardiac output. Arch Surg. (1991) 126:219–24. 10.1001/archsurg.1991.01410260109015 1992997

[10] Kimura S Yoshioka T Shibuya M Sakano T Tanaka R Matsuyama S. Indocyanine green elimination rate detects hepatocellular dysfunction early in septic shock and correlates with survival. Crit Care Med. (2001) 29:1159–63. 10.1097/00003246-200106000-00014 11395594

[11] Nesseler N Launey Y Aninat C Morel F Malledant Y Seguin P. Clinical review: the liver in sepsis. Crit Care (2012) 16:235. 10.1186/cc11381 23134597 PMC3682239

[12] Jacob A Zhou M Wu R Wang P. The role of hepatic cytochrome P-450 in sepsis. Int J Clin Exp Med. (2009) 2:203–11. 19918313 PMC2770183

[13] Kaner Z Ochayon DE Shahaf G Baranovski BM Bahar N Mizrahi M. Acute phase protein alpha1-antitrypsin reduces the bacterial burden in mice by selective modulation of innate cell responses. J Infect Dis. (2015) 211:1489–98. 10.1093/infdis/jiu620 25389308

[14] Harper PL Taylor FB DeLa Cadena A Courtney M Colman RW Carrell RW. Recombinant antitrypsin Pittsburgh undergoes proteolytic cleavage during E. coli sepsis and fails to prevent the associated coagulopathy in a primate model. Thromb Haemost. (1998) 80:816–821. 10.1055/s-0037-1615364 9843177

[15] Raasch M Rennert K Jahn T Peters S Henkel T Huber O. Microfluidically supported biochip design for culture of endothelial cell layers with improved perfusion conditions. Biofabrication 7:015013. 10.1088/1758-5090/7/1/015013 25727374

[16] Rennert K Steinborn S Groger M Ungerbock B Jank AM Ehgartner J. A microfluidically perfused three dimensional human liver model. Biomaterials (2015) 71:119–31. 10.1016/j.biomaterials.2015.08.043 26322723

[17] Gröger M Rennert K Giszas B Weiss E Dinger J Funke H. Monocyte-induced recovery of inflammation-associated hepatocellular dysfunction in a biochip-based human liver model. Sci Rep. (2016) 6:21868. 10.1038/srep21868 26902749 PMC4763209

[18] Gripon P Rumin S Urban S Le Seyec J Glaise D Cannie I. Infection of a human hepatoma cell line by hepatitis B virus. Proc Natl Acad Sci USA. (2002) 99:15655–60. 10.1073/pnas.232137699 12432097 PMC137772

[19] Jaffe EA Nachman RL Becker CG Minick CR. Culture of human endothelial cells derived from umbilical veins. Identification by morphologic and immunologic criteria. J Clin Invest. (1973) 52:2745–56. 10.1172/JCI107470 4355998 PMC302542

[20] Mosig S Rennert K Krause S Kzhyshkowska J Neunubel K Heller R. Different functions of monocyte subsets in familial hypercholesterolemia: potential function of CD14+ CD16+ monocytes in detoxification of oxidized LDL. FASEB J. (2009) 23:866–74. 10.1096/fj.08-118240 19001052

[21] Tukey JW Exploratory Data Analysis. Reading, MA: Addison-Wesley (1977).

[22] Bauer M Press AT Trauner M. The liver in sepsis: patterns of response and injury. Curr Opin Crit Care (2013) 19:123–127. 10.1097/MCC.0b013e32835eba6d 23448974

[23] Zamek-Gliszczynski MJ Xiong H Patel NJ Turncliff RZ Pollack GM Brouwer KL. Pharmacokinetics of 5 (and 6)-carboxy-2',7'-dichlorofluorescein and its diacetate promoiety in the liver. J Pharmacol Exp Ther. (2003) 304:801–9. 10.1124/jpet.102.044107 12538836

[24] Gonnert FA Recknagel P Hilger I Claus RA Bauer M Kortgen A. Hepatic excretory function in sepsis: implications from biophotonic analysis of transcellular xenobiotic transport in a rodent model. Crit Care (2013) 17:R67. 10.1186/cc12606 23574754 PMC4057165

[25] Levels JH Lemaire LC van den Ende AE van Deventer SJ van Lanschot JJ. Lipid composition and lipopolysaccharide binding capacity of lipoproteins in plasma and lymph of patients with systemic inflammatory response syndrome and multiple organ failure. Crit Care Med. (2003) 31:1647–53. 10.1097/01.CCM.0000063260.07222.76 12794399

[26] Luo G Guenthner T Gan LS Humphreys WG. CYP3A4 induction by xenobiotics: biochemistry, experimental methods and impact on drug discovery and development. Curr Drug Metab. (2004) 5:483–505. 10.2174/1389200043335397 15578943

[27] Thummel KE Shen DD Podoll TD Kunze KL Trager WF Bacchi CE. Use of midazolam as a human cytochrome P450 3A probe: II. Characterization of inter- and intraindividual hepatic CYP3A variability after liver transplantation. J Pharmacol Exp Ther. (1994) 271:557–66. 7965756

[28] Yan J Li S Li S. The role of the liver in sepsis. Int Rev Immunol. (2014) 33:498–510. 10.3109/08830185.2014.889129 24611785 PMC4160418

[29] Kjaergaard AG Dige A Krog J Tonnesen E Wogensen L. Soluble adhesion molecules correlate with surface expression in an *in vitro* model of endothelial activation. Basic Clin Pharmacol Toxicol. (2013) 113:273–9. 10.1111/bcpt.12091 23724832

[30] Wang HE Shapiro NI Griffin R Safford MM Judd S Howard G. Inflammatory and endothelial activation biomarkers and risk of sepsis: a nested case-control study. J Crit Care (2013) 28:549–55. 10.1016/j.jcrc.2012.11.002 23414982 PMC3814035

[31] Opal SM van der Poll T. Endothelial barrier dysfunction in septic shock. J Intern Med. (2015) 277:277–93. 10.1111/joim.12331 25418337

[32] Hordijk PL Anthony E Mul FP Rientsma R Oomen LC Roos D. Vascular-endothelial-cadherin modulates endothelial monolayer permeability. J Cell Sci. (1999) 112:1915–23. 10341210 10.1242/jcs.112.12.1915

[33] Baldwin AL Thurston G. Mechanics of endothelial cell architecture and vascular permeability. Crit Rev Biomed Eng. (2001) 29:247–78. 10.1615/CritRevBiomedEng.v29.i2.20 11417757

[34] Dejana E Spagnuolo R Bazzoni G. Interendothelial junctions and their role in the control of angiogenesis, vascular permeability and leukocyte transmigration. Thromb Haemost. (2001) 86:308–15. 10.1055/s-0037-1616228 11487019

[35] Desai TR Leeper NJ Hynes KL Gewertz BL. Interleukin-6 causes endothelial barrier dysfunction via the protein kinase C pathway. J Surg Res. (2002) 104:118–23. 10.1006/jsre.2002.6415 12020130

[36] Luo Y Radice GL. N-cadherin acts upstream of VE-cadherin in controlling vascular morphogenesis. J Cell Biol. (2005) 169:29–34. 10.1083/jcb.200411127 15809310 PMC2171890

[37] Wang D Yin Y Yao Y. Advances in sepsis-associated liver dysfunction. Burns Trauma (2014) 2:97–105. 10.4103/2321-3868.132689 27602369 PMC5012093

[38] Kellum JA Kong L Fink MP Weissfeld LA Yealy DM Pinsky MR. Understanding the inflammatory cytokine response in pneumonia and sepsis: results of the Genetic and Inflammatory Markers of Sepsis (GenIMS) Study. Arch Intern Med. (2007) 167:1655–63. 10.1001/archinte.167.15.1655 17698689 PMC4495652

[39] Mera S Tatulescu D Cismaru C Bondor C Slavcovici A Zanc V. Multiplex cytokine profiling in patients with sepsis. APMIS (2011) 119:155–63. 10.1111/j.1600-0463.2010.02705.x 21208283

[40] Essani NA Fisher MA Farhood A Manning AM Smith CW Jaeschke H. Cytokine-induced upregulation of hepatic intercellular adhesion molecule-1 messenger RNA expression and its role in the pathophysiology of murine endotoxin shock and acute liver failure. Hepatology (1995) 21:1632–9. 7768509

[41] Ledebur HC Parks TP. Transcriptional regulation of the intercellular adhesion molecule-1 gene by inflammatory cytokines in human endothelial cells. Essential roles of a variant NF-kappa B site and p65 homodimers. J Biol Chem. (1995) 270:933–43. 10.1074/jbc.270.2.933 7822333

[42] Doughty L Carcillo JA Kaplan S Janosky J. The compensatory anti-inflammatory cytokine interleukin 10 response in pediatric sepsis-induced multiple organ failure. Chest (1998) 113:1625–31. 10.1378/chest.113.6.1625 9631803

[43] Petrovic V Teng S Piquette-Miller M. Regulation of drug transporters during infection and inflammation. Mol Interv. (2007) 7:99–111. 10.1124/mi.7.2.10 17468390

[44] Recknagel P Claus RA Neugebauer U Bauer M Gonnert FA. *In vivo* imaging of hepatic excretory function in the rat by fluorescence microscopy. J Biophotonics (2012) 5:571–81. 10.1002/jbio.201100118 22271709

[45] Recknagel P Gonnert FA Westermann M Lambeck S Lupp A Rudiger A. Liver dysfunction and phosphatidylinositol-3-kinase signalling in early sepsis: experimental studies in rodent models of peritonitis. PLoS Med. (2012) 9:e1001338. 10.1371/journal.pmed.1001338 23152722 PMC3496669

[46] Jedlitschky G Hoffmann U Kroemer HK. Structure and function of the MRP2 (ABCC2) protein and its role in drug disposition. Expert Opin Drug Metab Toxicol. (2006) 2:351–66. 10.1517/17425255.2.3.351 16863439

[47] Stange J. Extracorporeal liver support. Organogenesis (2011) 7:64–73. 10.4161/org.7.1.14069 21343699 PMC3082035

[48] Fahrmayr C Konig J Auge D Mieth M Munch K Segrestaa J. Phase, I., and II metabolism and MRP2-mediated export of bosentan in a MDCKII-OATP1B1-CYP3A4-UGT1A1-MRP2 quadruple-transfected cell line. Br J Pharmacol. (2013) 169:21–33. 10.1111/bph.12126 23387445 PMC3632236

[49] Carcillo JA Doughty L Kofos D Frye RF Kaplan SS Sasser H. Cytochrome P450 mediated-drug metabolism is reduced in children with sepsis-induced multiple organ failure. Intensive Care Med. (2003) 29:980–4. 10.1007/s00134-003-1758-3 12698250

[50] Aitken AE Morgan ET. Gene-specific effects of inflammatory cytokines on cytochrome P450 2C, 2B6 and 3A4 mRNA levels in human hepatocytes. Drug Metab Dispos. (2007) 35:1687–93. 10.1124/dmd.107.015511 17576808 PMC2171046

[51] Morgan ET. Impact of infectious and inflammatory disease on cytochrome P450-mediated drug metabolism and pharmacokinetics. Clin Pharmacol Ther. (2009) 85:434–8. 10.1038/clpt.2008.302 19212314 PMC3139248

[52] Pape D Lehmann U Oellerich M Regel G. Multiple organ failure after severe trauma: predictable by the MEGX liver function test? Langenbecks Arch Chir Suppl Kongressbd (1996) 113:338–339. 9101869

[53] Aird WC. The role of the endothelium in severe sepsis and multiple organ dysfunction syndrome. Blood (2003) 101:3765–77. 10.1182/blood-2002-06-1887 12543869

[54] Zhang F Liu AL Gao S Ma S Guo SB. Neutrophil dysfunction in sepsis. Chin Med J. (2016) 129:2741–4. 10.4103/0366-6999.193447 27824008 PMC5126167

